# Risk of breast cancer in women after a salivary gland carcinoma or pleomorphic adenoma in the Netherlands

**DOI:** 10.1002/cam4.3598

**Published:** 2020-11-28

**Authors:** Matthijs H. Valstar, Michael Schaapveld, Esther C. van den Broek, Marie‐Louise F. van Velthuysen, Mischa de Ridder, Marjanka K. Schmidt, Boukje A.C. van Dijk, Alfons J.M. Balm, Ludi E. Smeele

**Affiliations:** ^1^ Department of Head and Neck Oncology and Surgery Netherlands Cancer Institute/Antoni van Leeuwenhoek Amsterdam The Netherlands; ^2^ Department of Oral and Maxillofacial Surgery Amsterdam University Medical Center University of Amsterdam Amsterdam The Netherlands; ^3^ Division of Psychosocial Research and Epidemiology Netherlands Cancer Institute/Antoni van Leeuwenhoek Amsterdam The Netherlands; ^4^ The Nationwide Network and Registry of Histo‐ and Cytopathology in the Netherlands (PALGA Houten The Netherlands; ^5^ Department of Pathology Erasmus Medical Centre Rotterdam The Netherlands; ^6^ Department of Radiation Oncology Leiden University Medical Center University of Leiden Leiden The Netherlands; ^7^ Division of Molecular Pathology Netherlands Cancer Institute/Antoni van Leeuwenhoek Amsterdam The Netherlands; ^8^ Netherlands Comprehensive Cancer Organization (IKNL Department of Research and Development Utrecht The Netherlands; ^9^ Department of Epidemiology University of Groningen University Medical Centre Groningen Groningen The Netherlands

**Keywords:** breast cancer, cancer risk factors, estrogen, head and neck cancer, pleomorphic adenoma, salivary gland cancer

## Abstract

Salivary and mammary gland tumors show morphological similarities and share various characteristics, including frequent overexpression of hormone receptors and female preponderance. Although this may suggest a common etiology, it remains unclear whether patients with a salivary gland tumor carry an increased risk of breast cancer (BC). Our purpose was to determine the risk of BC in women diagnosed with salivary gland carcinoma (SGC) or pleomorphic adenoma (SGPA). BC incidence (invasive and in situ) was assessed in two nationwide cohorts: one comprising 1567 women diagnosed with SGC and one with 2083 women with SGPA. BC incidence was compared with general population rates using standardized incidence ratio (SIR). BC risk was assessed according to age at SGC/SGPA diagnosis, follow‐up time and (for SGC patients) histological subtype. The mean follow‐up was 7.0 years after SGC and 9.9 after SGPA diagnosis. During follow‐up, 52 patients with SGC and 74 patients with SGPA developed BC. The median time to BC was 6 years after SGC and 7 after SGPA. The cumulative risk at 10 years of follow‐up was 3.1% after SGC and 3.5% after SGPA (95% Confidence Interval (95%CI) 2.1%–4.7% and 2.6%–4.6%, respectively). BC incidence was 1.59 times (95%CI 1.19–2.09) higher in the SGC‐cohort than expected based on incidence rates in the general population. SGPA‐patients showed a 1.48 times (95%CI 1.16–1.86) higher incidence. Women with SGC or SGPA have a slightly increased risk of BC. The magnitude of risk justifies raising awareness, but is no reason for BC screening.

## Graphical abstract

Salivary and mammary gland tumors share biological and epidemiological characteristics, suggesting common risk factors. Investigation of nationwide cohorts of patients with salivary gland cancer or pleomorphic adenoma with long‐term follow‐up data and complete cancer incidence information, highlights an increased risk of breast cancer in these patients.

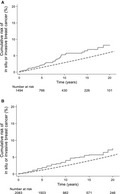

## 1 INTRODUCTION

Major salivary gland cancer (SGC) and salivary gland pleomorphic adenoma (SGPA) together constitute almost three quarters of all salivary gland tumors, and have a yearly incidence of respectively 0.7 and 4.9/100,000 person‐years in the Netherlands (European Standardized Rate).^1^, ^2^ Benign salivary gland tumors occur 6–7 times more frequently than malignant tumors and the SGPA, which accounts for two thirds of all benign salivary gland tumors, may occasionally show malignant transformation.^2^, ^3^, ^4^, ^5^, ^6^

Salivary and mammary gland tumors show morphological similarities and salivary gland‐like type tumors have been described in the breast.^7^, ^8^, ^9^, ^10^, ^11^ They also share other characteristics, including frequent overexpression of hormone receptors and in some histologies a female preponderance, most consistently in salivary gland adenoid cystic carcinoma and SGPA.^1^, ^2^, ^3^, ^12^, ^13^, ^14^, ^15^, ^16^

Whereas the larger amount of glandular breast tissue (next to hormonal, lifestyle, and genetic factors) puts women at higher risk of BC than men, gland volume does not explain the higher risk of a salivary gland tumor in women, since there are no gender differences in salivary gland size.^17^ Differential attitudes toward physical appearance or towards medical attention seeking behavior between males and females are unlikely to play a major role in explaining gender variation in incidence of SGC and SGPA, due to visibility of the tumor.^18^, ^19^, ^20^

Although the similarities may suggest a common etiology, it remains uncertain whether patients with a salivary gland tumor carry an increased risk of breast cancer (BC). Literature is inconclusive regarding whether risk is increased, and if so, to what extent and in which patients. A previous salivary gland tumor as a risk factor for BC has been reported in the literature, but studies are ambiguous, possibly due to the variation in sample size and inclusion criteria.^21^, ^22^, ^23^, ^24^, ^25^, ^26^, ^27^, ^28^ Support for a hormonal component in salivary gland cancer risk was earlier reported in an epidemiological study, and a hormonal influence could also have played a role in the recently reported higher risk of SGC in women after BC.^29^, ^30^

The objective of this study was to determine whether women diagnosed with a SGC or SGPA have an increased risk of developing BC in two nationwide population‐based cohorts with long term follow‐up and complete cancer incidence data.

## 2 MATERIALS AND METHODS

### 2.1 Cohorts

To assess the association between salivary gland tumors and BC risk in women, we used two nationwide Dutch registries. The Netherlands cancer registry (NCR) was used to establish a cohort including all incident malignant major salivary gland tumors (ICD‐O‐3 codes: C07: Parotid; C08: other and unspecified major salivary gland), diagnosed in the Netherlands between 1 January 1989 and 31 December 2014, without limitations regarding inclusion by age or previous other malignancies. All subsequent ductal in situ as well as invasive first BCs, registered in the NCR, which occurred until 31 December 2014 in this cohort were identified. The NCR receives its data mainly from the nationwide histopathology and cytopathology network and archive in The Netherlands (PALGA‐ in Dutch: Pathologisch Anatomisch Landelijk Geautomatiseerd Archief), but also from hospital discharge diagnosis registries (e.g. patients with a clinically or radiologically diagnosed SGC, who did not undergo biopsy or surgical treatment). Vital status and in situ and invasive breast cancer incidence were complete until 31 December 2014. Treatment details were reported and usually included surgery with post‐operative radiotherapy in selected cases. The latter typically consists of external beam radiotherapy, which is usually up to 70 Gray (Gy) on the tumor and 50 Gy on the neck, and has a horizontal direction and stays above the clavicle. For the SGPA cohort, we selected all women from a previously established SGPA cohort that included all Dutch pathologically confirmed incident SGPA diagnoses in the PALGA registry between 1 January 1992–31 December 2012 with (for logistical reasons to reduce the size of the cohort) a 5‐year interval.^2^ Therefore, all included SGPA patients were from the years 1992, 1997, 2002, 2007, and 2012. PALGA records all cytological and histological diagnoses, including those of benign diseases like SGPA, and has complete coverage of the Netherlands since 1991. PALGA is one of only a few national registries worldwide that include benign tumors, which allowed establishing our nationwide SGPA cohort.^2^, ^3^ The Palga registry does not contain standardized information on therapy. However, all patients in this cohort had a histological diagnose, thus had surgery. Radiotherapy is typically applied in selected recurrent SGPA cases. All subsequent DCIS as well as all invasive first BCs, registered in PALGA, which occurred until 31 December 2013 in this cohort were identified. Basal cell carcinoma and melanoma of the breast were excluded. Patients with BC before SGC or SGPA were excluded as well.

### 2.2 Statistical analysis

Time at risk started at date of SGC or SGPA diagnosis. For SGC follow‐up ended at the date of BC diagnosis, death, emigration, or last follow‐up, whichever came first. In SGPA patients, the expected number of cases and cumulative risk of BC were based on the cumulative follow‐up time. Since vital status is not registered in the PALGA registry, mortality among SGPA patients was imputed using gender‐, age‐, and calendar‐year specific life‐tables for the Dutch population, generating 50 imputed datasets, under the assumption that SGPA patients, as SGPA is a benign disease, had a similar life expectancy as the Dutch general population. Estimated cumulative risks and standard errors in each imputed dataset were subsequently pooled using Rubin's rule.

Expected BC incidence in the SGC cohort was estimated using the Hakulinen method.^31^ The Standardized Incidence Ratio (SIR) was calculated as the ratio between the observed and expected number of BC cases in both the SGC‐cohort and the SGPA‐cohort. In order to compare the observed BC incidence in our study population with the BC incidence among Dutch females from the general population, we used external reference rates. Using age‐specific (5‐year age groups) and calendar‐year specific BC incidence rates for the Dutch female population, we calculated the number of BCs we could have expected if our cohort would have had the same age‐specific BC incidence as the general population, based on the number of person‐years of follow‐up our women accrued in each 5‐year age group during each year of follow‐up. This method of using external data as reference data has been extensively used previously.^32^ Thus, the number of BC cases in both the SGC‐cohort and the SGPA‐cohort are compared with BC cases in a contemporized follow‐up period in contemporized age categories, in the Dutch population.

As a sensitivity analysis, the SIR was also determined for BCs occurring ≥3 months after the index salivary gland tumor, thereby excluding potential synchronous BC. In this analysis time at risk started at 93 days after SGC or SGPA diagnosis. Also, as a sensitivity analysis risk of invasive and in situ BC was evaluated separately. BC risk was also assessed and stratified for SGC/ SGPA age, follow‐up time and (in SGC patients) histological subtype. For SGC patients the 5 and 10‐year survival after BC was calculated, with a subgroup analysis for patients younger than 65 years. The 95% confidence intervals (95%CI) were calculated assuming a Poisson distribution for the observed number of events. Tests for homogeneity and trend of SIRs were performed using Poisson regression models based on collapsed person‐time data. Statistical analysis was performed using STATA 13 (StataCorp LP).

## 3 RESULTS

Our study included 3650 women: 1567 women with SGC and 2083 women with SGPA, translating into a yearly mean of 60 and 417 women respectively (Table 1; Table S2). The overall median age at diagnosis of the salivary gland tumor was 56 years (IQR 45–68). The median follow‐up was 7 years (IQR 2–12) after SGC. The median follow‐up after an SGPA diagnosis was 6 years (IQR 2–16). During follow‐up 52 SGC patients developed BC, of whom 46 (88%) had invasive and 6 (12%) in situ BC (Table 2; Table S3). Of the SGPA patients 74 developed BC, of whom 68 (92%) had invasive and 6 (8%) in situ BC. Overall, the median age at BC diagnosis was 63 years (IQR 51–74). The median interval between salivary gland tumor and BC diagnosis was 6 years in SGC (range 0–24; IQR 3–9) and 7 years (range 0–20; IQR 3–11) in SGPA. In SGC patients, the 5‐ and 10‐year survival rate after invasive BC was respectively 59.8% (95%CI 42.7–73.4%) and 42.0% (95%CI 24.9%–58.1%). For patients younger than 65 years, this was 78.9% (95%CI 52.4%–91.7%) and 70.1% (95%CI 40.5%–87.0%). The BC receptorstatus could be determined in most cases (Table 2).

**TABLE 1:** Population characteristics of the salivary gland carcinoma (SGC) or salivary gland pleomorphic adenoma (SGPA) with and without *subsequent* breast cancer (BC)

	SGC		SGPA		
	Subsequent BC (n = 52)	No subsequent BC (n = 1515)	BC (n = 74)		no BC (n = 2009)
Age at diagnosis, years^b^ (IQR)	57 (49–69)	63 (47–75)	50 (42–66)		48 (37–63)
Year of salivary tumor diagnose					
1989–1994	13	282	22		321
1995–2004	25	557	37		746
2005–2013	14	676	15		942
Histology SG tumor^a^, N(%)					
Adenoid cystic ca.	9 (17.3)	305 (20.1)			
Muco‐epidermoid ca.	9 (17.3)	227 (15)			
Acinic cell ca.	9 (17.3)	263 (17.4)			
Ca. ex Pleomorphic Adenoma	6 (11.5)	106 (7)			
Adenocarcinoma., NOS	7 (13.5)	204 (13.5)			
Squamous cell ca.	2 (3.8)	95 (6.3)			
Myo‐epithelial ca.	4 (7.7)	95 (6.3)			
Salivary duct ca.	1 (1.9)	29 (1.9)			
Other salivary gland ca.	5 (9.6)	190 (12.5)			
Pleomorphic adenoma			74 (100)		2009 (100)
Stage, N (%)			NA		NA
I	15 (28.8)	436 (28.8)			
II	10 (19.2)	226 (14.9)			
III	4 (7.7)	163 (10.8)			
IV	5 (9.6)	328 (21.7)			
Unknown/unavailable	18 (34.6)	362 (23.9)			
Treatment SG tumor N (%)					
Surgery with radiotherapy	27 (51.9)	881 (58.2)	74 (100.0)	2083 (100)	
Surgery only	13 (25)	359 (23.7)			
Radiotherapy only	3 (5.8)	91 (6)			
No therapy	3 (5.8)	76 (5)			
Surgery+radiotherapy+other	5 (9.6)	41 (2.7)			
Other	0 (0)	42 (2.8)			
Unknown/unavailabe	1 (1.9)	25 (1.7)			

Abbreviation: NA, not applicable.

^a^ Histology ICD‐O 3.1 Codes are shown in Table S2.

^b^ Median.

**TABLE 2:** Breast cancer (BC) histology and receptor status of patients with a salivary gland carcinoma (SGC) or salivary gland pleomorphic adenoma (SGPA) and *subsequent* BC

	BC after SGC (n = 52)	BC after SGPA (n = 74)
Year of BC diagnose, N (%)		
1989–1994	2 (3.8)	23 (31.1)
1995–2004	11 (21.2)	37 (50)
2005–2014	39 (75)	14 (18.9)
Age at diagnosis		
Median, years (IQR)	64 (57–76)	61 (50–69)
<50	6 (11.5)	17 (23)
50–69	25 (48.1)	38 (51.4)
>70	21 (40.3)	19 (25.7)
Histology BC^a^, N (%)		
*Invasive carcinoma*		
Lobular carcinoma	6 (11.5)	5 (6.7)
Ductal/Adeno carcinoma	39 (75)	57 (77)
Other BC (e.g. undiff. / NOS)	1 (1.9)	6 (8.1)
In situ carcinoma		
DCIS	6 (11.5)	6 (8.1)
Receptor status, N (%)		
ER+^b^	28 (53.8)	51 (68.9)
ER–	8 (15.4)	11 (14.9)
ER unknown	16 (30.8)	12 (16.2)
PR+^c^	22 (42.3)	39 (52.7)
PR–	11 (21.2)	19 (25.7)
PR unknown	19 (36.5)	16 (21.6)
Her2neu+^d^	6 (11.5)	8 (10.8)
Her2neu–	14 (26.9)	40 (54.1)
Her2neu unknown	32 (61.5)	26 (35.1)

Abbreviation: NA, not applicable.

^a^ Histology ICD‐O 3.1 Codes are shown in Table S3.

^b^ Estrogen receptor.

^c^ Progesteron receptor.

^d^ Human Epidermal growth factor Receptor 2.

### 3.1 Comparison with the general population

**FIGURE 1:**
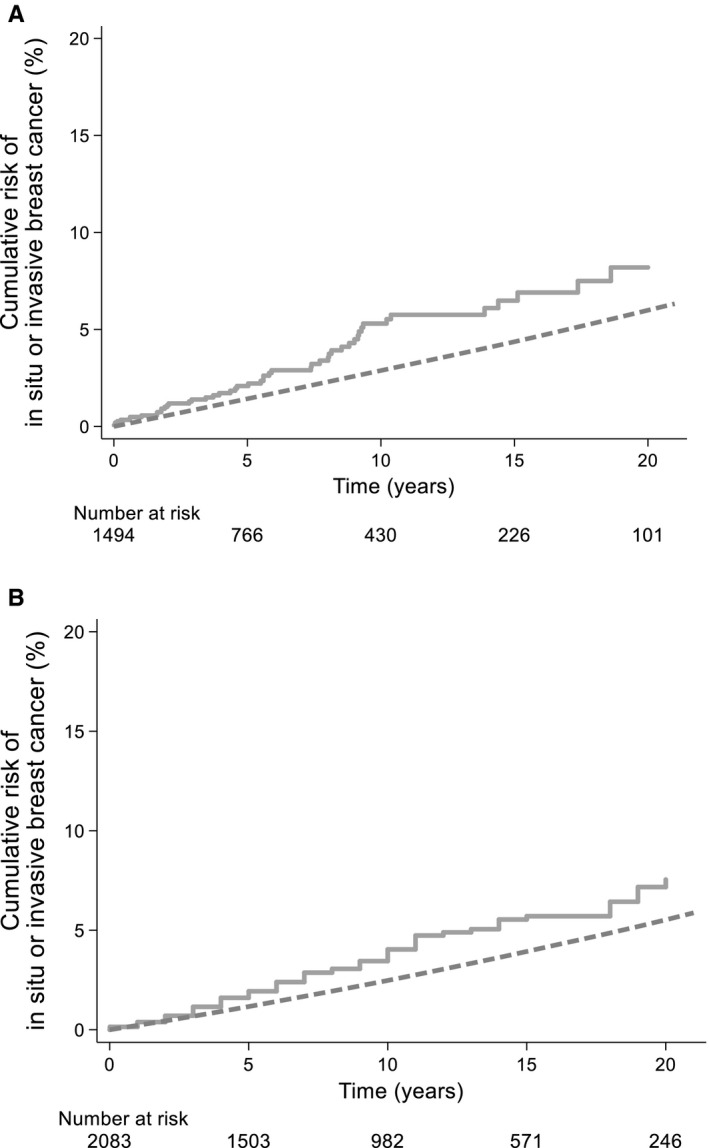
The cumulative risk of in situ and invasive breast cancer over a 20‐year period is increased after both salivary gland cancer (A) and pleomorphic adenoma (B), compared to the background population (dotted line)

The cumulative risk of BC in SGC patients was 5.3% (95% CI 3.8%–7.3%) at 10 and 8.2% (95% CI 5.8%‐11.5%) at 20 years, respectively (Table 3). The comparison to the expected risk based on the general population of 2.9% at 10 years and 6.0% at 20 years (dotted line) is made in Figure 1A. The cumulative risk of BC among SGPA patients was 3.5% (95% CI 2.6%–4.6%) at 10 years and 7.2% (95% CI 5.4%–9.4%) at 20 years, respectively (Table 3). In comparison, the expected cumulative BC risk, based on age‐matched incidence in the general population, was 2.2% at 10 and 5.2% at 20 years, respectively (Figure 1B). Overall, among SGC patients the incidence of BC was 1.59 times (95% CI 1.19–2.09) higher than expected based on general population rates. BC incidence was 1.48 times (95% CI 1.16–1.86) higher than expected among SGPA patients. The SIRs when risk of DCIS and invasive BC in the SGC‐cohort were estimated separately were similar (SIR 1.86 for in situ BC; 95% CI 0.68–4.04 and SIR 1.57 for invasive BC; 95% CI 1.14–2.09, respectively). SIRs for BC (invasive or in situ) did not vary with age at SGC diagnosis (SIR <50 years: 1.54, 95% CI 0.79–2.68; SIR 50–69 years: 1.69, 95% CI 1.10–2.48; SIR ≥70 years: 1.48, 95% CI 0.81–2.49, p‐heterogeneity = 0.91) nor did they change with follow‐up duration (SIR <10 years: 1.76, 95% CI 1.28–2.38; SIR 10–19 years: 0.92, 95% CI 0.37–1.89; SIR ≥20 years: 2.70, 95% CI 0.56–7.90, p‐heterogeneity = 0.19) or histological SGC subtype (Table 3). For SGPA patients, BC incidence did neither vary with age at SGPA diagnosis nor with follow‐up duration. A sensitivity analysis, showed similar risks after exclusion of synchronous BC (n = 4), defined as all BC diagnosed <3 months after the salivary gland tumor diagnosis; the SIR was 1.57; (95% CI 1.14–2.09) for SGC and 1.33 (95% CI 1.04–1.64) for SGPA, respectively.

**TABLE 3:** Standardized Incidence ratios (SIR), absolute excess risk (AER), and 20‐year cumulative risk of breast cancer following diagnosis of a salivary gland carcinoma (SGC), or a salivary gland pleomorphic adenoma (SGPA)

	SGC						SGPA					
	Events SGC	Person years	SIR	95%CI	AER	20‐year cum risk	Events SGPA	Person years^a^	SIR^b^	95%CI^b^	AER^b^	20‐year cum risk^b^
All	52	10945	1.59	1.19–2.09	17.7	8.2%	74	18852	1.48	1.16–1.86	12.8	7.2%
												
Age (years)												
<50	12	4369	1.54	0.79–2.68	9.6	3.2%	36	10797	1.85	1.30–2.56	15.3	6.7%
50–69	26	4056	1.69	1.10–2.48	26.2	12.4%	25	6222	1.06	0.69–1.57	2.4	6.4%
≥70	14	2520	1.48	0.81–2.49	18.1	16.2%	13	1833	1.87	1.00–3.20	33.1	11.5%
*P‐heterogeneity*			*0.91*						*0.07*			
Follow‐up (years)												
<10	42	8229	1.76	1.27–2.38	7.8	NA	53	13720	1.56	1.17–2.04	13.9	NA
10–19	7	2421	0.92	0.37–1.89	−2.6	NA	21	4896	1.39	0.86–2.12	12.0	NA
≥20	3	296	2.70	0.56–7.90	64.0	NA	0	235	0	0–4.31	−36.5	NA
*P‐trend*			*0.48*						*0.37*			
Histology												
Adenoid cystic carcinoma	9	2659	1.18	0.54–2.23	5.0	6.1%						
Muco‐epidermoid carcinoma	9	1868	1.96	0.90–3.72	23.6	7.0%						
Acinic cell carcinoma	9	2393	1.40	0.64–2.65	10.7	5.5%						
Carcinoma ex pleiomorphic adenoma	6	751	2.33	0.86–5.08	45.7	8.9%						
Adenocarcinoma, nos	7	1246	1.66	0.67–3.43	22.4	13.2%						
Carcinoma, other	12	2028	1.68	0.87–2.93	23.9	13.1%						
*P‐heterogeneity*			*0.81*									

Abbreviation: NA, not applicable.

^a^ Average number of person‐years over 50 imputed datasets.

^b^ SIR, AER, cumulative risks and confidence intervals based on pooled estimates.

In the SGC‐cohort, the absolute excess risk (AER) of DCIS and invasive BC combined was 17.7, in other words 17.7 excess BC for every 1000 women, each followed for 10 years. In the SGPA‐cohort the AER was 12.8 per 10,000 person‐years.

## 4 DISCUSSION

In this large and nationwide study with long‐term complete follow‐up, including 3650 women diagnosed with SGC and SGPA, the risk of developing a subsequent BC is moderately increased. This roughly 50% higher relative risk of BC can be better interpreted by mentioning absolute numbers in an example. For a 50‐year‐old woman, the risk to develop breast cancer in the next 10 years (until the age of 60) equals 2.9%.^33^ After a previous diagnosis with SGC or SGPA, this risk would be 4.3%.

In contrast to many previous studies on BC risk after a salivary gland tumor, the nationwide cohorts used in our study either consisted solely of malignant or of benign tumors. The high number of patients allowed unbiased estimation of relative and absolute risk in both type of lesions and gave rise to the possibility of subgroup analysis.

The lower age at diagnosis of second tumors in the SGPA‐cohort compared to the SGC‐cohort (Table 2), was in line with the lower age of the SGPA patients and earlier publications.^1^, ^2^ The increased BC risk after SGC or SGPA did not vary much with age at index tumor diagnosis nor with SGC histological subtype. The finding of an increased risk in patients diagnosed with BC ≥3 months after the salivary gland tumor, showed that the increased BC incidence was not likely based on surveillance bias caused by diagnostic evaluation of the index tumor.

Results of previous studies varied from no risk increase upto 8‐fold increased risks (Table 4). Many of these studies had methodological shortcomings which made comparison and generalization of these results difficult.^21^, ^22^, ^23^, ^24^, ^25^, ^26^, ^27^, ^28^ For instance, most studies did not provide a confidence interval for the risk estimates presented.^23^, ^24^, ^25^, ^26^, ^28^ The completeness of follow‐up may have been a problem in single institution cohorts.^21^, ^22^, ^28^ Missing a diagnosis of even one patient with a second primary BC, would have had a substantial negative impact on the estimated risk in these cohorts which included between 4 and 15 BC cases each. Also, there could be a referral bias in comprehensive cancer centers, because of inclusion of a higher proportion of patients with second or multiple primaries. Variation in BC incidence between the USA and Europe unlikely explains the high risk in two American studies.^21^, ^26^ Additionally, BC incidence rates in the SEER registry are generally lower than in European populations.^34^ A later conducted population based SEER study could not confirm the reported higher BC risk after an earlier SGC (SIR 1.07; 95% CI 0.72–1.53).^27^ This absence of an increased risk could have been caused by differences in inclusion, by including lesions of undefined histology (overall 25%) as a first tumor. These could have been metastases of, for example, squamous cell carcinoma of the skin of the face or skull to the parotid, which could have attenuated a higher risk of BC.

**TABLE 4:** A comparison of publications investigating breast cancer (BC) incidence in women after diagnosis of a salivary gland tumour (SGT)

Ref, year	Women (n)	SGT (M/B)	Years at risk	Observed BC	Expected BC	O/E (SIR)	*p* value	95% CI
1968^21^	396	M	1652	7	0.9	7.8	0.00004	NR
1969^22^	297	M	3033	4	4.0	1.0	NA	NA
1972^23^	349	M	2443	8	4.2	1.9	0.6	NR
1977^24^	453	M + B	2315	6	2.6	2.3	<0.05	NR
1983^25^	367	M	2868	7	5.4	1.3	NR	0.5–2.7
1984^26^	190	M + B	629	4	0.83	4.8	0.01	NR
1999^27^	1718	M^a^	10,789	30	28	1.07	NR	0.72–1.53
2005^28^	439	M + B	3382	15	5.93	2.5	0.003	1.4–4.2
Current	2083	B	18,852	74	50	1.48	NR	1.16–1.86
2019	1567	M	10,995	52	32.6	1.59	NR	1.19–2.09

Abbreviations: B, benign; M, malignant; NA, not applicable; NR, not reported.

^a^ 25% undetermined / mixed histology and 13% squamous cell carcinoma.

The increased risk of BC after a salivary gland tumor in our nationwide cohorts, is of similar magnitude as some of the known risk factors, such as alcohol intake or use oral contraceptives (Table S1) and is remarkable. It could theoretically be caused by several mechanisms.

### 4.1 Common endocrine mechanism and common environmental / lifestyle factors

A positive association between (high levels of) endogenous estrogens or estrogen exposure and risk of various female cancers (breast, ovarian and endometrial cancer) has been consistently described in the epidemiological literature.^35^, ^36^, ^37^, ^38^ Estrogens are thought to cause this increased cancer risk via cell proliferation, DNA‐damage (as a result of estradiol metabolism to genotoxic metabolites) and inhibition of cell repair mechanisms.^39^ Estrogen exposure may have similar effects on salivary glandular tissue, which also expresses estrogen receptors. It could therefore be that women with high endogenous estrogen levels (e.g. in post‐menopausal high BMI) or exposure (e.g. in postmenopausal hormone replacement therapy or use of oral contraceptives) have an increased risk of SGC or SGPA.^40^, ^41^, ^42^, ^43^, ^44^ If estrogen indeed has an effect on both risk of SGC and risk of SGPA, this could also explain the reported female preponderance of SGC and SGPA. In the literature, generally only a small portion of salivary gland tumors show ER‐overexpression based on immunohistochemistry, possibly because mainly ER‐α and not ER‐β expression status has been assessed.^45^, ^46^, ^47^, ^48^, ^49^, ^50^, ^51^ Nevertheless, although ER‐β expression in salivary gland tumors was only reported in a limited number of series, ER‐β was overexpressed in 27 of 38 (71%) patients in a series of adenoid cystic carcinomas and in 57 of 80 (73%) in a series of salivary duct carcinomas.^16^, ^52^

In SGPA and adenoid cystic carcinoma, the slight, but relevant difference in incidence between males and females (female to male incidence ratio of 1.4:1 and 1.2:1, respectively) and the overexpression of estrogen β receptor (ER‐β), compared to normal salivary gland, may suggest a role for the proliferative influence of estrogens in tumorigenesis.^2^, ^14^, ^53^ Similar considerations have been mentioned in earlier reports of experimental anti‐estrogen treatment for SGC in the literature.^8^, ^54^, ^55^ From the above, it could be concluded it is a realistic possibility that estrogens play a role in the association between salivary gland tumors and BC.

### 4.2 Salivary gland tumor treatment or metastasis

It is unlikely that SGC of SGPA treatment contributes to an increased BC risk since locoregional treatment does not affect breast tissue. In external beam radiotherapy for SGC, the breast is not an organ at risk due to the direct dose, although scattered radiation could in theory add to the risk of primary breast cancer. This dose can be calculated.^56^ After radiotherapy for SGC in the earlier mentioned example of a 50‐year old female patient, this estimated additional lifetime risk is 0.3%, *for all cancers*. So, scattering does not contribute substantially to the risk of BC.

An increased BC risk due to SGC chemotherapy (depending on histology, but mostly cisplatin based) is not very likely.^57^, ^58^ Solitary metastasis of SGC to the breast is not very likely. The SGPA is known to almost never metastasize at all. Also, if there were cases, these would probably have been recorded in the pathology database or cancer registry not as BC but as metastasized salivary gland tumor.

### 4.3 Common genetic susceptibility

Germline mutations that could account for the occurrence of both a salivary gland tumor and BC in the same woman have not been identified yet, and the literature on this topic is limited. One retrospective study including 5754 proven or likely carriers from 187 *BRCA1 or BRCA2* positive pedigrees, reported three SGCs.^59^ Although the authors reported an increased SGC incidence, a 95% CI was not provided. Mersch and colleagues did not find any SGC in 1072 patients with a *BRCA1* or *BRCA2* mutation, who had received genetic counselling.^60^ In a study of 268 patients with SGPA, upon evaluation for *BRCA1*, one had a *BRCA1* mutation (who earlier also had BC).^61^ Other forms of genetic predisposition, that is, based on (multiple) single‐nucleotide polymorphisms (SNPs) have not been reported. Although SGPA is characterized by a chromosomal translocation that causes activation of the pleomorphic adenoma gene 1 (PLAG1) on chromosome 8q12, germline mutations have not yet been identified.^62^, ^63^, ^64^, ^65^, ^66^

### 4.4 Limitations

Although the results of this study suggest that salivary gland tumors and BC share a common (hormonal) etiological factor, further investigation will be needed to provide more insight in the underlying mechanism. It is still unclear whether affected women who develop both a salivary gland tumor and BC are, for example, more susceptible for the effect of estrogens, have higher blood levels or have had more estrogen exposure. Data on BC risk factors were unavailable as these are not routinely collected by the Dutch cancer registry.

Person time after an SGPA diagnosis was not available and imputed assuming these patients had a similar life expectancy as the general Dutch population. SGPA does carry a very small risk of malignant transformation of approximately 0.15%, which may lead to a slightly higher mortality than that of the general population.^2^ We may thus have overestimated the follow‐up in the SGPA cohort, which would have resulted in slight overestimation of the expected number of BC cases,^2^ and subsequently an underestimation of the SIR for BC. Follow‐up of SGC patients is very complete, with information on dates of death or migration provided by linkage with the Dutch nationwide population registry, and near complete information on BCs occurring in this cohort, available from the same source.

In summary, there are some indications that suggest an endocrine mechanism behind the increased incidence of BC in salivary gland tumours. Data on a possible genetic background are insufficient. Sequencing of the cohorts for possible germline mutations might provide further clues regarding genetic predisposition, but this would require very large cohorts.

### 4.5 Clinical implications

Women with a SGC or SGPA have a moderately increased risk of BC, compared to women in the general population. The relative risk for BC in patients with SGC or SGPA (together almost 75% of salivary gland tumors) is of similar magnitude as in patients who have one of various classical BC risk factors. However, the magnitude of the relative risk is no reason for recommending an intensified follow‐up schedule. Nevertheless, our study should raise awareness of a slightly increased risk of developing BC among female patients diagnosed with SGC or SGPA and their treating physicians.

## AUTHOR CONTRIBUTIONS

Matthijs Valstar: conceptualization, data curation, formal analysis, investigation, methodology, project administration, resources, validation, visualization, writing ‐ original draft. Michael Schaapveld: data curation, formal analysis, investigation, methodology, resources, validation, visualization, writing—review and editing. Esther van den Broek: conceptualization, data curation, formal analysis, investigation, methodology, project administration, resources, writing—review and editing. Marie‐Louise van Velthuysen: conceptualization, data curation, writing—review and editing. Mischa de Ridder: conceptualization, data curation, methodology, project administration, writing—review and editing. Marjanka Schmidt: interpretation of data, writing—review and editing. Boukje van Dijk: data curation, methodology, resources, validation, visualization, writing—review and editing. Alfons Balm: conceptualization, methodology, writing—review and editing, supervision. Ludi Smeele: conceptualization, methodology, writing—review and editing, supervision.

## ETHICAL APPROVAL

Not applicable; all patient information that was provided to the authors, was anonimized beforehand by the two national registries, after the research protocol was approved by their scientific boards.

## Supporting information

Supplementary Material

## Data Availability

Research data are available upon reasonable request.
