# Supplementary material for: Risk of breast cancer in women after a salivary gland carcinoma or pleomorphic adenoma in the Netherlands
Source: Cancer Med. 2020 Nov 28;10(1):424–34. doi: 10.1002/cam4.3598 (PMC7826476; doi:10.1002/cam4.3598)
Supplement: Supplementary file 1 — Supplementary Material [file CAM4-10-424-s001.docx]

**Supplementary table 1 (online only):**

**Risk factors and relative risk (RR) for breast cancer (BC)**

| **Risk Factor** | **RR** |  | **Risk factor** | **RR** |
| --- | --- | --- | --- | --- |
| Increased age (>45 vs <45)^1,2^  BRCA1 / 2 mutations^1,2^  Geographic region^1^  High density on mammogram^3^  Atypical benign breast disorders^1,2,4,5^  Previous radiotherapy chest or axilla <age 40^6–8^  Previous BC or DCIS^4^  Age at 1^st^ childbirth >35 vs <20^1,2^  Postmenopausal high bone density^1^  Diethylstilbestrolin pregnancy^2^ | >10  6-8  5-10  4-6  4-5  3-20  2->4  2  2-3.5  2 |  | Late menopause, >54 yr^1,2,4^  Nulliparity^1,2,4^  HRT>10 yr^1^  Alcohol intake (2-5 U/D)^9–11^  Oral contraceptives (recently / past)^1,12^  Early age of menarge, age<11 ^1,2,4^  Inactivity vs physical exercise 5x/wk ^13,14^  In vitro fertilisation^15–17^  Weight: premenopausal BMI>35;  postmenopausal BMI>35^2^ | ≤ 2  <2  1.4-3  1.2-1.5  1.2 –2.4  1-6  1-3  0.85  0.7  2 |

Adapted from Oncoline^82^

**Supplementary table 2 (online only):**

**ICD-O-3.1 Histology code grouping of salivary gland tumors**^19^

| **Histology** | **ICD-code** |
| --- | --- |
| Adenoid cystic carcinoma | 8200 |
| Muco-epidermoid carcinoma | 8430 |
| Acinic cell carcinoma | 8550, 8551 |
| Squamous cell carcinoma | 8070, 8071, 8072, 8074, 8075, 8076, 8078, 8083 |
| Adenocarcinoma NOS | 8140, 8190, 8201, 8230, 8260, 8440, 8450, 8471, 8480, 8481, 8490, 8503, 8525, 8574 |
| Carcinoma ex pleiomorphic adenoma | 8022, 8940, 8941 |
| Myo-epithelial carcinoma | 8562, 8982 |
| Salivary duct carcinoma | 8500 |
|  |  |
| Other salivary gland carcinomas |  |
| 8000 = neoplasm NOS | 8240 = carcinoidtumor |
| 8001 = malignant tumor cells NOS | 8246 = neuro-endocrine carcinoma NOS |
| 8010 = carcinoma NOS | 8247 = merckel cell carcinoma |
| 8012 = large cell carcinoma NOS | 8249 = atypical carcinoid |
| 8013 = neuro-endocrine carcinoma large cell | 8290 = oxyphilic adenocarcinoma |
| 8020 = undifferentiated carcinoma | 8310 = clear cell adenocarcinoma |
| 8021 = anaplastic carcinoma | 8501 = comedocarcinoma |
| 8031 = large cell carcinoma | 8502= secretory carcinoma of breast |
| 8032 = spindle cell carcinoma | 8510 = medullary adenocarcinoma |
| 8033 = sarcomatoid carcinoma | 8560 = adenosquamous carcinoma |
| 8041 = small cell carcinoma | 8575 = metaplastic carcinoma |
| 8082 = lympho-epithelial carcinoma | 8974 = sialoblastoma |
| 8094 = basosquamous carcinoma | 8980 = carcinoma sarcoma NOS |
| 8147 = basalceladeno carcinoma | 9990 = no microscopic confirmation |
|  |  |

Of 1,567 SGC patients, three were registered with a non-histology confirmed diagnosis of SGC in the Dutch Cancer Registry, based on the clinical behavior and radiological aspect. A reason for this can be e.g. refusal of cytology (FNA) and surgical biopsy and treatment. None of these patients had a subsequent BC, and no BC diagnosis was based only on clinical behavior or radiological aspect.

**Supplementary table 3 (online only):**

**ICD-O-3.1 Histology code grouping of breast cancers(BC)**^19^

| **BC histology in SGC cases** | **ICD-code** | **N** |
| --- | --- | --- |
| Adenocarcinoma | 8140 | 4 |
| Papillary carcinoma | 8260 | 1 |
| Mucinous adenocarcinoma | 8480 | 1 |
| Intraductal carcinoma | 8500 | 36 (incl. 5 DCIS) |
| Intraductal papillary adenocarcinoma | 8503 | 1 |
| Medullary adenocarcinoma | 8510 | 1 |
| Lobulary carcinoma | 8520 | 6 |
| Infiltrating ductal and lobular carcinoma | 8522 | 1 |
| Paget disease with intraductal carcinoma | 8543 | 1 (incl. 1 DCIS) |

| **BC histology in SGPA cases** | **ICD-code** | **N** |
| --- | --- | --- |
| Adenocarcinoma | 8140 | 27 |
| Papillary carcinoma | 8260 | 0 |
| Mucinous adenocarcinoma | 8480 | 1 |
| Intraductal carcinoma | 8500 | 34 (incl. 5 DCIS) |
| Intraductal papillary adenocarcinoma | 8503 | 0 |
| Medullary adenocarcinoma | 8510 | 0 |
| Lobulary carcinoma | 8520 | 6 (incl. 1 DCIS) |
| Infiltrating ductal and lobular carcinoma | 8522 | 0 |
| Paget disease with intraductal carcinoma | 8543 | 0 |
| Carcinoma NOS | 8010 | 5 |
| Carcinoma undiff. NOS | 8020 | 1 |
